# Time-restricted feeding during the inactive phase vs. *ad libitum* feeding: diet-dependent effects on weight and metabolic markers in male Wistar rats

**DOI:** 10.3389/fnut.2026.1968504

**Published:** 2026-10-06

**Authors:** Ladan Hajhamidiasl, Murat Baş, Ozgur Yilmaz, Samed Ozer, Salim Yilmaz, Sedat Arslan

**Affiliations:** 1 Department of Nutrition and Dietetics, Faculty of Health Sciences, Acibadem Mehmet Ali Aydinlar University, Istanbul, Türkiye; 2 Department of Nutrition and Dietetics, Graduate School of Health Sciences, Acibadem Mehmet Ali Aydinlar University, Istanbul, Türkiye; 3 Animal Application and Research Center, Acibadem Mehmet Ali Aydinlar University, Istanbul, Türkiye; 4 Department of Healthcare Management, Faculty of Health Sciences, Acibadem Mehmet Ali Aydinlar University, Istanbul, Türkiye; 5 Department of Healthcare Management, Graduate School of Health Sciences, Acibadem Mehmet Ali Aydinlar University, Istanbul, Türkiye; 6 Department of Nutrition and Dietetics, Bursa Uludag University, Bursa, Türkiye

**Keywords:** *ad libitum*, circadian rhythm, high-fat diet, intermittent fasting, time-restricted feeding

## Abstract

**Background/objectives:**

In recent years, intermittent fasting has become a popular method for preventing and treating obesity and chronic diseases. This study explored the metabolic and inflammatory effects of inactive-phase (Day) time-restricted feeding (TRF) vs. *ad libitum* feeding (Ad) in rats under high-fat (HF) and standard (S) diet for 8 weeks.

**Methods:**

Four groups (TRF + HF, TRF + S, Ad + S, Ad + HF; *n* = 7 each) were monitored for temporal changes in key biomarkers, including TNF-α, IL-6, LPS, insulin, glucose, and lipid profiles, and body weight. Baseline-adjusted two-way ANCOVA was used to evaluate the main effects of Diet and Feeding pattern and their interaction, with complementary multivariate analyses performed as sensitivity analyses.

**Results:**

Body weight was significantly associated with both Diet and Feeding pattern after baseline adjustment, with the greatest observed weight gain in Ad + HF and the smallest in TRF + S. TNF-α showed a significant Diet × Feeding interaction, characterized by marked reductions in both TRF groups and little change in Ad + HF. Lipid metabolism exhibited distinct interactions between diet type and feeding regimens. Total cholesterol levels increased in both *ad libitum*-fed groups, whereas a reduction was observed in the TRF + S group. Triglyceride responses also showed pronounced treatment effects, with the TRF + HF group demonstrating the greatest decrease over the intervention period. IL-6, LPS, insulin, glucose, and LDL-C did not display significant group × time interactions.

**Conclusions:**

Overall, inactive-phase time-restricted feeding was associated with lower weight gain, favorable changes in selected lipid parameters, and a reduction in TNF-α. No significant Diet × Feeding interactions were observed for IL-6 or LPS, indicating that the inflammatory findings were specific to TNF-α rather than reflective of a generalized anti-inflammatory effect. However, because food intake was not quantitatively measured, these differences cannot be attributed to meal timing independently of possible differences in total energy intake.

## 1 Introduction

Obesity represents a widespread global health issue (1, 2) and arises from a prolonged imbalance in which energy intake exceeds energy expenditure (3–5). Sustained positive energy balance leads to adipocyte hypertrophy, followed by adipocyte dysfunction, hyperglycemia, dyslipidemia, ectopic fat accumulation, chronic low-grade inflammation, and impaired insulin sensitivity (6–12). These alterations contribute to the development of conditions such as type 2 diabetes mellitus and cardiovascular disease (13, 14). The rising prevalence of obesity and metabolic syndrome has intensified interest in dietary strategies to mitigate excessive weight gain. Although prolonged calorie restriction with adequate nutrient intake has been shown to reduce body weight and obesity-associated complications (15), maintaining such regimens over long periods is challenging (16). These challenges are further compounded by compensatory changes in appetite and energy expenditure (17–19), as well as poor long-term adherence (20–23). Intermittent fasting (IF) has emerged as a widely adopted weight-loss approach due to its simplicity and documented metabolic benefits. IF is increasingly recognized as a practical and effective strategy for promoting weight loss and improving metabolic health indicators (24).

There are various forms of IF, including time-restricted feeding (TRF), whole-day fasting, and alternate-day fasting, each with varying durations and frequencies (24). In TRF, the time frame during which individuals can eat is restricted, without necessarily reducing their overall calorie intake. Within a designated window, such as 3–4, 7–9, or 10–12 h, individuals are free to consume their regular diet as they desire. This method creates extended periods of fasting each day, which may yield various health benefits (25).

Consistently limiting food access to specific time frames each day through TRF may induce food-anticipatory activity, influenced by the internal circadian clock (26).

In humans, aligning meals with active (daytime) periods through time-restricted feeding enhances glucose regulation, lipid profiles, and blood pressure without necessarily requiring calorie restriction (26–28). Likewise, in rodents, feed restriction to the active (dark) phase synchronizes feeding–fasting cycles with circadian rhythms of metabolism, leading to better glucose tolerance, reduced adiposity, and lower inflammatory signaling compared with *ad libitum* feeding (29). These positive effects are attributed to enhanced alignment between central and peripheral clocks, increased insulin sensitivity, and rhythmic activation of lipid oxidation pathways (30). The alignment of food intake with the organism's endogenous circadian phase critically determines all these outcomes. In nocturnal rodents, feeding during the light period (inactive phase) induces circadian misalignment, whereby the central suprachiasmatic nucleus remains synchronized to the light–dark cycle, while peripheral metabolic clocks in tissues such as muscle, adipose tissue, and liver shift toward the feeding schedule (31). This misalignment disrupts the rhythmic expression of clock-controlled genes that regulate lipid and glucose metabolism, potentially leading to insulin resistance, dyslipidemia, oxidative stress, and inflammation (30).

Evidence from both human and animal studies indicates that time-restricted eating can confer metabolic benefits regardless of whether food intake is aligned with the circadian active phase (32, 33). In humans, comparisons of early and late time-restricted eating show that both approaches can improve metabolic outcomes, although via partially distinct mechanisms. Early time-restricted eating, which is more closely aligned with endogenous circadian rhythms, tends to produce stronger improvements in insulin sensitivity and glucose regulation, whereas late time-restricted eating—despite circadian misalignment—has been associated with reductions in body weight and fat mass, as well as improvements in lipid and inflammatory profiles (32). Similarly, in rodent models, both early (active-phase) and late (inactive-phase) time-restricted HF feeding attenuate weight gain and improve metabolic parameters compared with *ad libitum* feeding. Although circadian-aligned feeding generally yields more pronounced effects on glucose homeostasis and circadian metabolic regulation, inactive-phase time-restricted feeding has also been shown to reduce adiposity, underscoring the substantial contribution of prolonged daily fasting to metabolic health even under conditions of circadian misalignment (33).

Nevertheless, evidence from Ramadan fasting—a natural model of inactive-phase time-restricted feeding in humans—indicates that daily fasting intervals can confer metabolic benefits, as demonstrated by a study reporting reductions in body weight and inflammatory markers together with improvements in lipid profiles and insulin sensitivity, despite predominant nighttime food intake (34). These human studies support the idea that the fasting–feeding cycle may provide metabolic advantages even when eating occurs during the circadian inactive phase. In addition to circulating cytokines, lipopolysaccharide (LPS) may provide an indirect marker of metabolic endotoxemia associated with impaired intestinal barrier function. High-fat feeding can promote intestinal permeability and facilitate the translocation of bacterial products such as LPS into the circulation, where LPS may contribute to low-grade systemic inflammation through pathways including TLR4/NF-κB signaling (35, 36). Therefore, circulating LPS was included in the present study to explore whether inactive-phase TRF was associated with changes in this inflammation-related pathway. Based on these findings, the present study aimed to examine the protective effects of TRF, even when applied during the inactive phase in male Wistar rats fed either a high-fat or a standard diet. Specifically, food access was restricted to an 8-h window, which matches the rats' inactive (light) phase (09:00–17:00), consistent with the nighttime feeding pattern observed in humans during Ramadan fasting. Four experimental groups were established based on feeding regimen (TRF or *ad libitum*) and diet type (high-fat or standard). The main objective was to determine whether an 8-week inactive-phase TRF could reduce body weight gain and alter metabolic and inflammatory markers, including lipid profile, glucose, insulin, TNF-α, interleukin (IL)-6, and lipopolysaccharide (LPS). By evaluating these outcomes, this study aimed to characterize the metabolic responses associated with inactive-phase TRF while recognizing that the design does not permit direct assessment of circadian alignment or separation of fasting-duration effects from feeding-phase effects. This approach provides preliminary insight into how meal timing could influence obesity and metabolic disease risk.

## 2 Materials and methods

### 2.1 Animals

Male Wistar Albino rats (8–12 weeks old, weighing 250–300 g, *n* = 7/group) were housed and studied at the Experimental Animals Application and Research Center of Acibadem Mehmet Ali Aydinlar University (ACU-DEHAM) under controlled conditions (12 h/12 h light/dark cycles [lights on from 07:00 to 19:00, lights off from 19:00 to 07:00], temperature 21 °C ± 2 °C, humidity 65–70%). Animals were housed in standard polypropylene cages measuring 425 × 265 × 180 mm. Each experimental group consisted of seven rats distributed across four cages, with two rats housed in each of three cages and one rat housed individually in the fourth cage. Routine environmental enrichment (e.g., bedding material and nesting opportunities) was provided in accordance with institutional animal care guidelines.

Only male rats were used, as they tend to gain weight faster than female rats and therefore allow for larger effect sizes within an 8-week study period (37). All protocols were approved by the Local Ethics Committee for Animal Experiments of Acibadem Mehmet Ali Aydinlar University (ACU-HADYEK 2022/17) on 05.01.2022.

*A priori* sample size calculation was performed prior to the start of the experiment. The required sample size was determined *a priori* using G^*^Power 3.1.9.7 for a mixed-design ANOVA (4 groups × 2 time points) targeting the group × time interaction. Assuming a medium-to-large effect size (f = 0.40), an alpha level of 0.05, a correlation among repeated measures of 0.50, and a nonsphericity correction ε = 1, the analysis indicated that a total of 28 rats was required to achieve 90% statistical power (actual power = 0.92). Accordingly, 28 rats (n = 7 per group) were included in the study.

### 2.2 Experimental design

A total of 28 rats were randomly allocated into four experimental groups (*n* = 7 per group) using a computerized randomization algorithm to ensure balanced group assignment and minimize selection bias. All animals were acclimatized to the housing conditions prior to the initiation of experimental procedures. Investigators responsible for daily animal care and feeding were aware of group assignments due to the nature of the dietary interventions. However, all outcome assessments were performed in a blinded manner. Specifically, biochemical and ELISA analyses were conducted by a researcher who was unaware of group identities until all data collection was completed, thereby reducing the risk of detection bias.

The diet protocols are described in Table 1.

**Table 1:** Diet protocols of the study groups.

Group	Diet	Name
Group 1 (*n* = 7)	Time-restricted access to a high-fat diet for 8 h only during the inactive (light) phase	TRF + HF
Group 2 (*n* = 7)	Time-restricted access to a standard chow diet for 8 h only during the inactive (light) phase	TRF + S
Group 3 (*n* = 7)	Continued *ad libitum* feeding of the standard rodent chow	Ad + S
Group 4 (*n* = 7)	Continued *ad libitum* feeding of the high-fat diet	Ad + HF

### 2.3 Diets

Rats in groups 2 and 3 were fed a standard chow diet, whereas those in groups 1 and 4 were fed a high-fat diet. The high-fat diet was used to induce a diet-induced obesity model. In the high-fat diet, the principal added fat sources were palm oil (175 g) and corn oil (25 g) per formulation. Groups 3 and 4 were fed *ad libitum*, while groups 1 and 2 had time-restricted access to food for 8 h during the light (inactive) phase, from 09:00 to 17:00. Throughout the study, all rats had *ad libitum* access to water. All animals completed the 8-week experimental period, and no abnormal clinical signs, distress, or other health concerns were observed.

The energy and macronutrient compositions of the high-fat and standard chow diets (ARDEN Feed Industry Research & Experimental Trade Company, Istanbul, Türkiye) are presented in Table 2.

**Table 2:** Energy and macronutrient compositions of the high-fat and standard chow diets.

Section	Component	High-fat diet	Standard chow diet
Energy density	Energy (kcal/g)	4.73	3.10
Macronutrient distribution (kcal%)	Protein	20	24
	Carbohydrate	35	58
	Fat	45	18
Macronutrient composition (g%)	Protein	24	17.5
	Carbohydrate	41	47
	Fat	24	6
Other components (%)	Crude fiber	4.7	5.0
	Ash	4.5	5.1
Ingredients (g)	Casein (30 mesh)	200	
	Methionine	3	
	Maltodextrin	100	
	Sucrose	172.8	
	Cellulose (BW200)	50	
	Corn oil	25	
	Palm oil	175	
	Mineral mix (S10026)	10	
	DiCalcium phosphate	13	
	Calcium carbonate	5.5	
	Potassium citrate, 1 H_2_O	16.5	
	Vitamin mix (V10001)	10	
	Choline bitartrate	2	
	FD&C red dye #40	0.05	

Food access was controlled by providing and removing the diet tray daily; however, food consumption was monitored only qualitatively, and quantitative assessment of daily food intake (in grams) was not performed.

### 2.4 Body weight monitoring

Body weight was measured at baseline and weekly throughout the 8-week experimental period. Absolute weight gain was defined as the difference between Week 8 and baseline body weight, and percentage weight gain was calculated relative to baseline body weight.

### 2.5 Measurement of serum metabolites

To ensure reproducibility and minimize circadian-related variability, blood sampling was conducted under strictly standardized fasting and timing conditions. For the TRF groups, which had access to food only during the light phase from 09:00 to 17:00, blood sampling at week 8 was performed after a standardized 6-h fasting period at 23:00 (6 h after the end of the feeding window). To ensure comparable metabolic conditions, rats in the *ad libitum* groups were also deprived of food after 17:00 and fasted for the same 6-h period before sampling; thus, all week-8 blood samples were collected at 23:00 across all groups. In addition, to avoid circadian inconsistencies between baseline and final measurements, all baseline samples were also collected at 23:00 following a 6-h fast, ensuring identical fasting duration and circadian timing at both time points. Blood samples were collected at two points. At baseline, 2 ml of blood was collected from the jugular vein under isoflurane inhalation anesthesia. At the end of the 8-week intervention, 2 ml blood was collected by cardiac puncture under isoflurane anesthesia.

All samples were analyzed in a single analytical session. Before the session, the serum samples were thawed overnight at 4 °C. Following the final blood collection, the animals were euthanized under anesthesia in accordance with the approved animal study protocol. Serum concentrations of TNF-α, IL-6, LPS, and insulin were measured using enzyme-linked immunosorbent assay (ELISA) kits (catalog numbers: TNF-α: E0764Ra; IL-6: E0135Ra; LPS: E0756Ra; insulin: E-EL-R2466) following the manufacturer's instructions. Absorbance was read using a BIO-TEK ELX800 microplate reader, and washing steps were performed with the BIO-TEK ELX50 strip washer. Fasting glucose and lipid parameters—catalog numbers: glucose (OttoBC142), total cholesterol (OttoBC135), HDL-C (OttoBC144), LDL-C (OttoBC145), and triglycerides (OttoBC155)—were determined using enzymatic colorimetric assay kits on a MINDRAY BS-400 automated chemistry analyzer.

Two composite lipid indices were calculated to provide additional cardiovascular risk assessment. Non-HDL cholesterol was calculated as total cholesterol minus HDL cholesterol (Non-HDL = TC – HDL). The TC/HDL ratio was calculated as total cholesterol divided by HDL cholesterol (TC/HDL ratio = TC / HDL).

### 2.6 Statistical analysis

All analyses were conducted using Python 3.13. The normality of continuous variables was evaluated using the Shapiro–Wilk test, and homogeneity of variances was assessed using Levene's test. The study followed a 2 × 2 factorial design with Diet [high-fat (HF) vs. standard (S)] and Feeding pattern [time-restricted feeding (TRF) vs. *ad libitum* (Ad)] as between-subject factors. The primary inferential analysis was a two-way ANCOVA for each outcome, with the Week 8 value as the dependent variable, Diet and Feeding pattern as fixed between-subject factors, and the corresponding baseline value as a covariate. Type III sums of squares were used to evaluate the main effects of Diet and Feeding pattern and the Diet × Feeding interaction. Thus, the primary ANCOVA model was a baseline-adjusted factorial analysis and not a repeated-measures ANCOVA. This approach was selected because (i) it adjusts for pre-existing group differences—particularly relevant given significant baseline differences in TNF-α (*p* = 0.018) and triglycerides (*p* = 0.006); (ii) it improves statistical power by reducing residual variance; and (iii) it provides unbiased estimates of treatment effects when baseline values correlate with post-intervention outcomes. ANCOVA assumptions—including residual normality, homogeneity of variances, linearity between the covariate and outcome, and homogeneity of regression slopes—were examined for each outcome. Weight, TNF-α, glucose, and HDL-C satisfactorily met all assessed assumptions. For IL-6, LPS, insulin, LDL-C, and triglycerides, some departures from normality were observed; however, variance homogeneity and the other model assumptions were considered acceptable. Given the balanced group sizes (*n* = 7 per group), the factorial ANCOVA was retained for these outcomes, with complementary sensitivity analyses used to assess the robustness of the findings.

This approach was employed as a sensitivity analysis for three reasons: (i) linear models provide coefficient estimates (B) with 95% confidence intervals that allow direct interpretation of effect magnitudes in original measurement units, whereas ANCOVA primarily yields F-statistics and *p*-values; (ii) coefficient estimates facilitate interpretation of effect magnitudes across different outcomes; and (iii) concordance between ANCOVA and linear model results strengthens confidence in the reported findings by demonstrating robustness across analytical approaches. As a complementary sensitivity analysis, a repeated-measures MANOVA using Pillai's trace was conducted to examine the overall multivariate pre–post pattern across the measured biomarkers. This analysis was considered supportive; the primary inferential conclusions were based on the baseline-adjusted factorial ANCOVA described above. Within-group pre–post changes were examined using paired *t*-tests for normally distributed differences and Wilcoxon signed-rank tests for non-normal data. *Post hoc* pairwise comparisons for significant main effects and interactions were conducted using Bonferroni correction (α = 0.0083). Effect sizes were reported as partial eta-squared (η^2^p) derived from Type III sum of squares using the formula η*p* = *SS*_*effect*_/(*SS*_*effect*_ + *SS*_*residual*_), with thresholds of 0.01, 0.06, and 0.14 representing small, medium, and large effects, respectively. Adjusted marginal means and 95% confidence intervals were reported for mean differences between factor levels. To assess the robustness of findings, supplementary analyses included Pearson correlations between baseline values and change scores to evaluate regression to the mean, Cohen's d effect sizes for within-group changes, and sensitivity analyses excluding potential outliers. Statistical significance was set at *p* < 0.05.

## 3 Results

The results indicated distinct patterns in weight gain, inflammatory markers, and metabolic parameters between the treatment conditions.

Inflammatory markers showed divergent treatment responses (Figure 1, Table 3). TNF-α levels decreased substantially in TRF groups (−40% in TRF + HF; −30% in TRF + S) and moderately in Ad + S (−19%), whereas Ad + HF maintained stable levels (+ 1%). IL-6 responses varied, with significant reductions in TRF + S (−31%) and Ad + HF (−21%), while Ad + S showed a slight increase (+ 11%). LPS levels decreased in all groups (−42% to −46%), with Ad + HF showing a smaller reduction (−21%).

**Figure 1:**
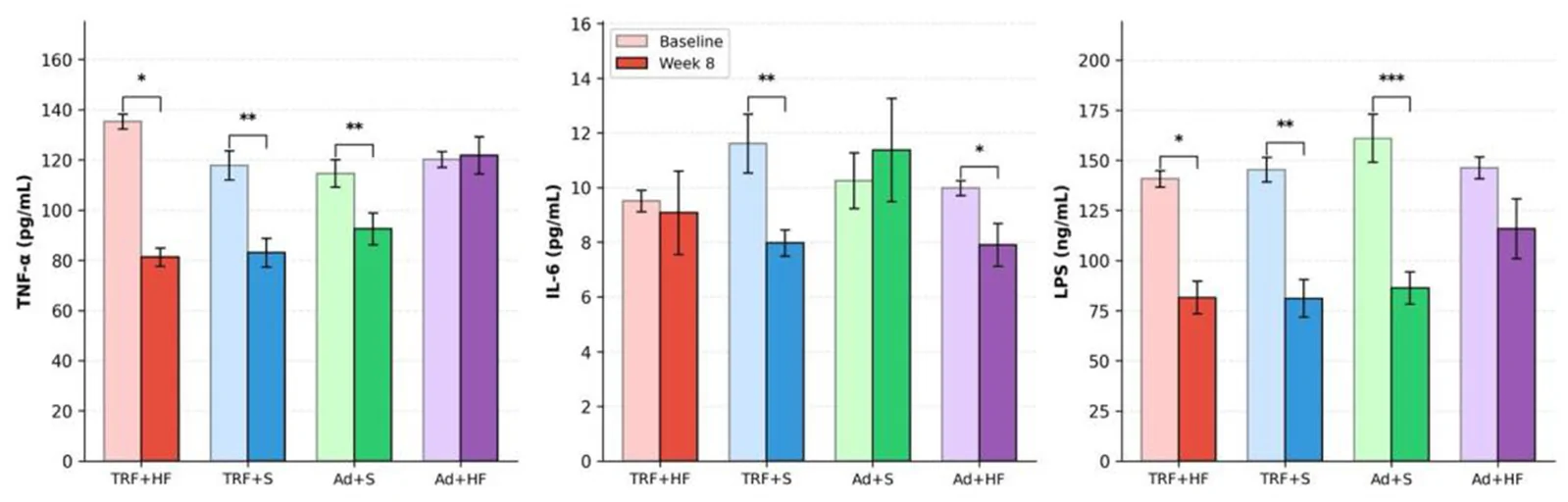
Changes in inflammatory biomarkers from baseline to Week 8 across experimental groups. Bars represent group means, and error bars represent the standard error of the mean (SEM). Lighter bars represent baseline values and darker bars represent Week 8 values. Asterisks indicate significant within-group changes from baseline to Week 8 (*p < 0.05, **p < 0.01, ***p < 0.001) based on paired *t*-tests or Wilcoxon signed-rank tests, as appropriate. TRF, time-restricted feeding; Ad, *ad libitum*; HF, high-fat diet; S, standard diet.

**Table 3:** Baseline and Week 8 values and within-group changes across experimental groups.

Variable	Measurement	TRF + HF	TRF + S	Ad + S	Ad + HF
Weight (g)	Baseline	433.5 ± 15.6	449.5 ± 25.6	422.6 ± 35.0	428.1 ± 36.6
	Endpoint	497.1 ± 37.0	469.0 ± 27.2	480.9 ± 45.3	562.9 ± 62.4
	Within *p*	W = 0.00, *p =* 0.016^*^	*t =* 2.52, *p =* 0.025^*^	W = 0.00, *p =* 0.016^*^	*t =* 11.28, *p < * 0.001^***^
Glucose (mg/dL)	Baseline	98.4 ± 13.9	97.4 ± 7.1	97.7 ± 8.9	99.0 ± 13.4
	Endpoint	112.7 ± 22.2	106.3 ± 13.3	101.6 ± 11.6	118.3 ± 8.1
	Within *p*	*t =* 1.44, *p =* 0.201	*t =* 1.54, *p =* 0.173	*t =* 0.65, *p =* 0.538	*t =* 3.46, *p =* 0.013^*^
Insulin (mIU/ml)	Baseline	1.2 ± 0.1	1.1 ± 0.2	1.1 ± 0.1	1.1 ± 0.1
	Endpoint	1.0 ± 0.2	0.9 ± 0.2	0.9 ± 0.3	0.9 ± 0.2
	Within *p*	W = 5.00, *p =* 0.156	*t =* 1.57, *p =* 0.169	*t =* 1.47, *p =* 0.192	W = 1.00, *p =* 0.031^*^
Cholesterol (mg/dL)	Baseline	72.6 ± 9.1	73.3 ± 16.9	58.3 ± 10.9	72.1 ± 14.1
	Endpoint	76.0 ± 9.3	66.6 ± 13.1	72.1 ± 9.9	88.6 ± 15.7
	Within *p*	*t =* 0.74, *p =* 0.487	*t =* 2.10, *p =* 0.080	W = 0.00, *p =* 0.016^*^	*t =* 2.35, *p =* 0.057
HDL (mg/dL)	Baseline	28.4 ± 5.1	25.4 ± 6.4	21.2 ± 7.2	27.1 ± 6.9
	Endpoint	47.2 ± 8.9	39.2 ± 8.1	42.1 ± 6.4	52.6 ± 12.1
	Within *p*	*t =* 6.07, *p < * 0.001^***^	*t =* 7.51, *p < * 0.001^***^	W = 0.00, *p =* 0.016^*^	*t =* 7.59, *p < * 0.001^***^
LDL (mg/dL)	Baseline	13.6 ± 4.0	14.3 ± 3.3	14.1 ± 2.0	17.3 ± 5.0
	Endpoint	15.9 ± 3.5	11.3 ± 2.9	16.4 ± 5.5	18.0 ± 4.2
	Within *p*	*t =* 1.05, *p =* 0.332	W = 1.50, *p =* 0.047^*^	W = 5.00, *p =* 0.281	*t =* 0.29, *p =* 0.781
Triglyceride (mg/dL)	Baseline	121.6 ± 33.4	165.1 ± 30.9	89.4 ± 41.0	125.1 ± 35.6
	Endpoint	39.9 ± 11.4	75.3 ± 15.2	40.6 ± 14.0	90.9 ± 38.2
	Within *p*	*t =* 8.33, *p < * 0.001^***^	*t =* 7.74, *p < * 0.001^***^	W = 0.00, *p =* 0.016^*^	W = 7.00, *p =* 0.297
Non-HDL (mg/dL)	Baseline	44.2 ± 8.5	47.9 ± 14.0	37.1 ± 6.1	45.0 ± 10.5
	Endpoint	28.8 ± 6.4	27.4 ± 8.7	30.0 ± 7.2	36.0 ± 9.4
	Within *p*	*t =* 3.52, *p =* 0.012^*^	*t =* 4.21, *p =* 0.006^**^	W = 2.00, *p =* 0.047^*^	*t =* 1.89, *p =* 0.107
TC/HDL ratio	Baseline	2.7 ± 0.5	3.0 ± 0.7	2.9 ± 0.5	2.8 ± 0.6
	Endpoint	1.7 ± 0.3	1.7 ± 0.3	1.7 ± 0.2	1.8 ± 0.4
	Within *p*	*t =* 5.92, *p < * 0.001^***^	*t =* 6.84, *p < * 0.001^***^	*t =* 8.41, *p < * 0.001^***^	*t =* 3.12, *p =* 0.021^*^
TNF-α (pg/ml)	Baseline	135.2 ± 7.8	117.8 ± 15.4	114.6 ± 14.5	120.1 ± 8.2
	Endpoint	81.2 ± 9.7	83.0 ± 14.9	92.5 ± 16.8	121.8 ± 19.6
	Within *p*	W = 0.00, *p =* 0.016^*^	*t =* 3.34, *p =* 0.003^**^	*t =* 4.06, *p =* 0.007^**^	*t =* 0.19, *p =* 0.858
IL-6 (pg/ml)	Baseline	9.5 ± 1.0	11.6 ± 2.9	10.2 ± 2.7	10.0 ± 0.7
	Endpoint	9.1 ± 4.0	8.0 ± 1.3	11.4 ± 5.0	7.9 ± 2.1
	Within *p*	W = 13.00, *p =* 0.938	*t =* 3.76, *p =* 0.009^**^	W = 13.00, *p =* 0.938	*t =* 2.69, *p =* 0.036^*^
LPS (ng/ml)	Baseline	140.7 ± 10.7	145.3 ± 16.1	160.9 ± 31.7	146.2 ± 14.3
	Endpoint	81.5 ± 21.6	81.1 ± 24.8	86.2 ± 21.2	115.8 ± 39.4
	Within *p*	W = 1.00, *p =* 0.031^*^	W = 0.00, *p =* 0.016^*^	*t =* 7.15, *p < * 0.001^***^	*t =* 1.72, *p =* 0.137

Post-hoc: Bonferroni-corrected pairwise comparisons due to uniform directional changes across all groups. Significance: ^*^p < 0.05, ^**^p < 0.01, ^***^p < 0.001. Effect size: η^2^*p* > 0.14 = large effect.

Metabolic parameters demonstrated diet- and feeding-specific effects. Insulin levels decreased modestly across all groups (−11% to −20%) (Figure 2). Cholesterol responses diverged: TRF + S showed a decrease (−9%) while *ad libitum* groups increased (+ 23% to + 24%). HDL increased substantially in all groups, with the largest gains in *ad libitum* groups (+ 94% to + 99%). Triglycerides showed marked reductions in TRF groups (−54% to −67%) compared to *ad libitum* groups (−27% to −55%). Glucose levels increased in all groups except Ad + S, with Ad + HF showing the highest increase (+20%).

**Figure 2:**
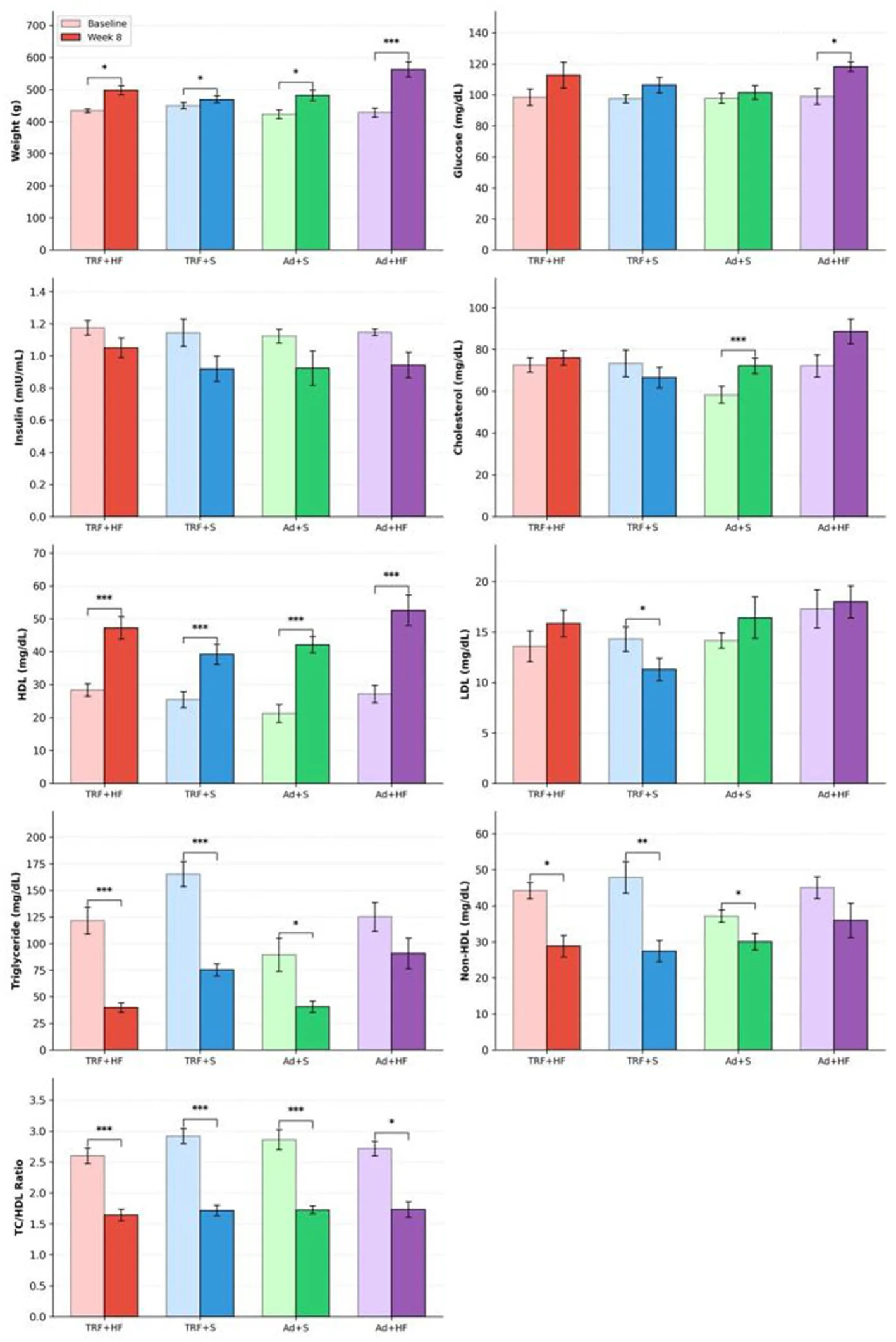
Changes in body weight and metabolic parameters from baseline to Week 8 across experimental groups. The figure presents body weight, glucose, insulin, total cholesterol, HDL-C, LDL-C, triglycerides, non-HDL cholesterol, and the TC/HDL ratio. Bars represent group means, and error bars represent the standard error of the mean (SEM). Lighter bars represent baseline values and darker bars represent Week 8 values. Asterisks indicate significant within-group changes from baseline to Week 8 (*p < 0.05, **p < 0.01, ***p < 0.001) based on paired *t*-tests or Wilcoxon signed-rank tests, as appropriate. TRF, time-restricted feeding; Ad, *ad libitum*; HF, high-fat diet; S, standard diet.

Body weight increased in all groups over the 8-week intervention, but the magnitude of gain differed across groups (Figure 3). Mean body weight increased from 433.5 ± 15.6 to 497.1 ± 37.0 g in TRF + HF (+ 63.6 g; + 14.7%), from 449.5 ± 25.6 to 469.0 ± 27.2 g in TRF + S (+ 19.5 g; + 4.3%), from 422.6 ± 35.0 to 480.9 ± 45.3 g in Ad + S (+ 58.2 g; + 13.8%), and from 428.1 ± 36.6 to 562.9 ± 62.4 g in Ad + HF (+ 134.8 g; + 31.5%). Thus, the greatest observed weight gain occurred in Ad + HF, whereas TRF + S showed the smallest increase. The baseline-adjusted two-way ANCOVA revealed significant main effects of both Diet and Feeding pattern on body weight (Diet: *p* = 0.003, η^2^*p* = 0.330; Feeding: *p* = 0.005, η^2^*p* = 0.290), whereas the Diet × Feeding interaction was not significant (*p* = 0.193, η^2^*p* = 0.073; Table 4).

**Figure 3:**
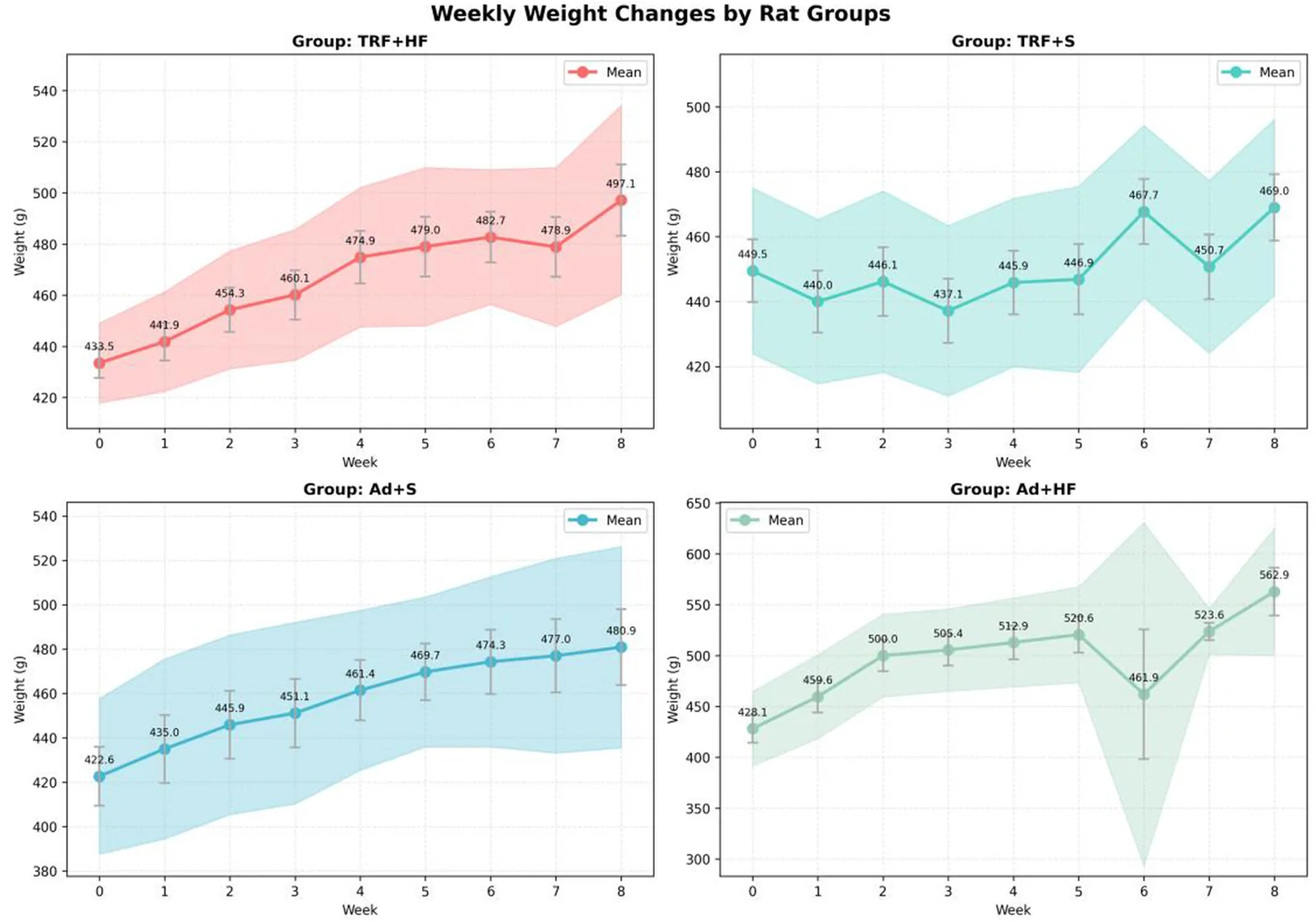
Temporal changes in body weight across experimental groups over the 8-week intervention. Each panel displays the weekly mean body weight for one experimental group. Shaded areas represent the standard deviation (SD), whereas vertical error bars at each time point represent the standard error of the mean (SEM). TRF + HF, time-restricted feeding with high-fat diet; TRF + S, time-restricted feeding with standard diet; Ad + S, *ad libitum* with standard diet; Ad + HF, *ad libitum* with high-fat diet.

**Table 4:** Two-way ANCOVA results for diet type and feeding pattern effects on metabolic outcomes and inflammation markers.

Variables	TRF + HF (*n* = 7)	TRF + S (*n* = 7)	Ad + S (*n* = 7)	Ad + HF (*n* = 7)	Diet (HF vs. S)			Feeding (Ad vs. TRF)			Interaction	
	Δ (%)	Δ (%)	Δ (%)	Δ (%)	F (*p*)	η^2^*p*	Δ [95% CI]	F (*p*)	η^2^*p*	Δ [95% CI]	F (*p*)	η^2^p
Weight (g)	63.6 (14.7%)	19.5 (4.3%)	58.2 (13.8%)	134.8 (31.5%)	11.33 (0.003)^**^	0.330	48.13 [18.55, 77.72]	9.39 (0.005)^**^	0.290	45.44 [14.76, 76.11]	1.80 (0.193)	0.073
Glucose (mg/dL)	14.3 (14.5%)	8.9 (9.1%)	3.9 (3.9%)	19.3 (19.5%)	0.64 (0.433)	0.027	6.44 [−10.24, 23.12]	0.34 (0.565)	0.015	−4.71 [−21.39, 11.96]	0.81 (0.376)	0.034
Insulin (mIU/ml)	−0.1 (−10.6%)	−0.2 (−19.6%)	−0.2 (−17.8%)	−0.2 (−17.7%)	1.66 (0.211)	0.067	0.15 [−0.09, 0.38]	0.00 (0.950)	0.000	−0.01 [−0.24, 0.23]	0.51 (0.484)	0.022
Cholesterol (mg/dL)	3.4 (4.7%)	−6.7 (−9.2%)	13.9 (23.8%)	16.4 (22.8%)	3.10 (0.091)	0.119	9.80 [−1.71, 21.32]	4.91 (0.037)^*^	0.176	13.46 [0.89, 26.02]	0.01 (0.937)	0.000
HDL (mg/dL)	18.8 (66.4%)	13.7 (54.0%)	20.9 (99.0%)	25.5 (93.8%)	1.95 (0.175)	0.078	5.24 [−2.52, 13.00]	3.40 (0.078)	0.129	7.03 [−0.86, 14.91]	0.01 (0.930)	0.000
LDL (mg/dL)	2.3 (16.8%)	−3.0 (−21.0%)	2.3 (16.2%)	0.7 (4.1%)	4.29 (0.050)^*^	0.157	4.67 [0.01, 9.32]	5.28 (0.031)^*^	0.187	5.16 [0.52, 9.81]	1.13 (0.298)	0.047
Triglyceride (mg/dL)	−81.7 (−67.2%)	−89.9 (−54.4%)	−48.9 (−54.6%)	−34.3 (−27.4%)	7.35 (0.012)^*^	0.242	−36.66 [−64.64, −8.68]	5.44 (0.029)^*^	0.191	−36.85 [−69.54, −4.17]	18.91 (< 0.001)^***^	0.451
Non–HDL (mg/dL)	−15.4 (−34.9%)	−20.4 (−42.7%)	−7.1 (−19.1%)	−9.0 (−20.1%)	0.06 (0.814)	0.002	1.18 [−9.06, 11.42]	0.14 (0.710)	0.006	2.07 [−9.29, 13.42]	0.49 (0.492)	0.021
TC/HDL Ratio	−1.0 (−36.8%)	−1.2 (−41.3%)	−1.1 (−39.7%)	−1.0 (−36.3%)	0.23 (0.638)	0.010	−0.07 [−0.36, 0.23]	0.01 (0.942)	0.000	0.01 [−0.27, 0.29]	0.15 (0.698)	0.007
TNF-alpha (pg/ml)	−54.0 (−39.9%)	−34.8 (−29.5%)	−22.1 (−19.3%)	1.6 (1.4%)	0.26 (0.618)	0.011	−4.90 [−24.97, 15.16]	1.40 (0.248)	0.058	10.10 [−7.53, 27.72]	7.14 (0.014)^*^	0.237
IL-6 (pg/ml)	−0.4 (−4.5%)	−3.6 (−31.3%)	1.1 (11.0%)	−2.1 (−20.8%)	0.04 (0.849)	0.002	0.38 [−3.69, 4.45]	2.40 (0.135)	0.094	2.93 [−0.98, 6.85]	2.18 (0.154)	0.086
LPS (ng/ml)	−59.2 (−42.1%)	−64.2 (−44.2%)	−74.7 (−46.4%)	−30.4 (−20.8%)	0.00 (0.999)	0.000	0.02 [−31.41, 31.45]	0.16 (0.692)	0.007	6.33 [−26.36, 39.02]	1.73 (0.202)	0.070

Values represent mean change from baseline with percent change in parentheses [absolute change (%)]. Baseline and endpoint group means (SD) are reported in Table 3. Analyses were conducted using two-way ANCOVA with baseline as covariate (Type III SS). Reference categories: Diet = Standard (S), Feeding = Time-Restricted Feeding (TRF). Positive Δ indicates higher endpoint values for HF diet (vs. S) or *Ad libitum* feeding (vs. TRF). Non-HDL cholesterol = Total cholesterol – HDL; TC/HDL Ratio = Total cholesterol / HDL. TRF = Time-restricted feeding; Ad = *Ad libitum*; HF = High-fat diet; S = Standard diet. η^2^*p* = partial eta-squared; Δ = adjusted mean difference with 95% confidence interval. ^*^p < 0.05, ^**^p < 0.01, ^***^p < 0.001.

The primary inferential analyses were performed using baseline-adjusted two-way ANCOVA, with Diet and Feeding pattern as between-subject factors and the corresponding baseline value as a covariate. Significant main effects of Diet and Feeding pattern were observed for body weight (Diet: *p* = 0.003, η^2^*p* = 0.330; Feeding: *p* = 0.005, η^2^*p* = 0.290), whereas the Diet × Feeding interaction was not significant (*p* = 0.193). For triglycerides, significant effects of Diet (*p* = 0.012, η^2^*p* = 0.242) and Feeding pattern (*p* = 0.029, η^2^*p* = 0.191), together with a significant Diet × Feeding interaction (*p* < 0.001, η^2^*p* = 0.451), were observed. TNF-α also showed a significant Diet × Feeding interaction (*p* = 0.014, η^2^*p* = 0.237), whereas neither main effect was significant. Feeding pattern showed significant main effects on total cholesterol (*p* = 0.037, η^2^*p* = 0.176) and LDL-C (*p* = 0.031, η^2^*p* = 0.187). No significant Diet × Feeding interactions were observed for glucose, insulin, total cholesterol, HDL-C, LDL-C, non-HDL cholesterol, TC/HDL ratio, IL-6, or LPS.

The baseline-adjusted two-way ANCOVA revealed significant main effects of both Diet and Feeding pattern on body weight, with the Ad + HF group showing the greatest observed increase and the TRF + S group the smallest. The Diet × Feeding interaction for body weight was not significant (Table 4).

Lipid metabolism revealed differential responses across groups. Total cholesterol demonstrated a significant interaction, with *ad libitum* groups showing increases and TRF + S exhibiting a decrease. HDL significantly increased in all groups, with *ad libitum* groups showing nearly double the baseline values. Triglycerides exhibited a strong interaction effect (*p* < 0.001), with TRF + HF showing the greatest reduction (−67%), followed by TRF + S (−54%) and Ad + S (−55%), whereas Ad + HF showed the smallest reduction (−27%), which did not reach within-group significance (*p* = 0.297).

Among inflammatory markers, TNF-α showed a substantial interaction, characterized by marked reductions in TRF groups while Ad + HF showed no significant change. No significant Diet × Feeding interactions were observed for IL-6, LPS, insulin, glucose, or LDL-C (all *p* > 0.05). The combination of TRF and standard diet appeared most effective in preventing weight gain and improving lipid profiles, whereas Ad + HF showed the least favorable metabolic outcomes (Table 3).

High-fat diet (relative to standard diet) demonstrated significant main effects on body weight (*p* = 0.003), LDL (*p* = 0.050), and triglycerides (*p* = 0.012), with high-fat diet groups showing greater weight gain compared to standard diet groups. *Ad libitum* feeding (relative to TRF) independently influenced body weight (*p* = 0.005), total cholesterol (*p* = 0.037), LDL (*p* = 0.031), and triglycerides (*p* = 0.029), with *ad libitum* groups showing greater weight gain and less favorable lipid profiles compared to time-restricted feeding groups. Notably, significant diet × feeding interactions emerged for triglyceride (*p* < 0.001) and TNF-α (*p* = 0.014), indicating that the effects of these factors on lipid and inflammatory markers were modulated by their combination (Table 4).

Composite lipid indices showed uniform improvements across all groups (Tables 3, 4). Non-HDL cholesterol decreased in all groups, with TRF groups showing larger reductions (−35% to −43%) compared to *ad libitum* groups (−19% to −20%). The TC/HDL ratio improved uniformly across all groups (−37% to −41% reductions), indicating enhanced cardiovascular risk profiles. However, two-way ANCOVA revealed no significant main effects of diet or feeding pattern, nor any interaction effects, for either composite index, suggesting these improvements occurred independent of experimental conditions.

Two-way ANCOVA with baseline adjustment revealed distinct contributions of diet type and feeding pattern to metabolic outcomes. Diet demonstrated significant main effects on body weight, LDL, and triglycerides, with high-fat diet groups showing greater weight gain compared to standard diet groups. Feeding pattern independently influenced body weight, total cholesterol, LDL cholesterol, and triglycerides, with time-restricted feeding groups demonstrating attenuated weight gain and improved lipid profiles relative to *ad libitum* groups. Notably, significant diet × feeding interactions emerged for triglyceride and TNF-α, indicating that the effects of feeding pattern on these inflammatory and lipid markers were modulated by diet composition (Table 4).

To provide more clinically relevant assessments of atherogenic risk, composite lipid indices were calculated and are presented in Figure 2 and Table 4. Non-HDL cholesterol decreased from baseline to week 8 in all groups, with TRF groups showing larger reductions (−35% to −43%) compared to *ad libitum* groups (−19% to −20%). Similarly, the TC/HDL ratio improved uniformly across all groups (−37% to −41% reductions). However, two-way ANCOVA revealed no significant main effects of diet or feeding pattern, nor any interaction effects, for either composite index, indicating uniform cardiovascular risk improvements independent of experimental conditions.

To visualize the interaction patterns identified in the ANCOVA analyses, interaction plots and individual trajectory visualizations were generated for selected outcomes to illustrate group-specific patterns and individual pre–post trajectories (Figure 4). The interaction plots (left column) display diet × feeding effects on change scores (Table 4), while the spaghetti plots (right column) show individual trajectories from baseline to week 8 (Table 3).

**Figure 4:**
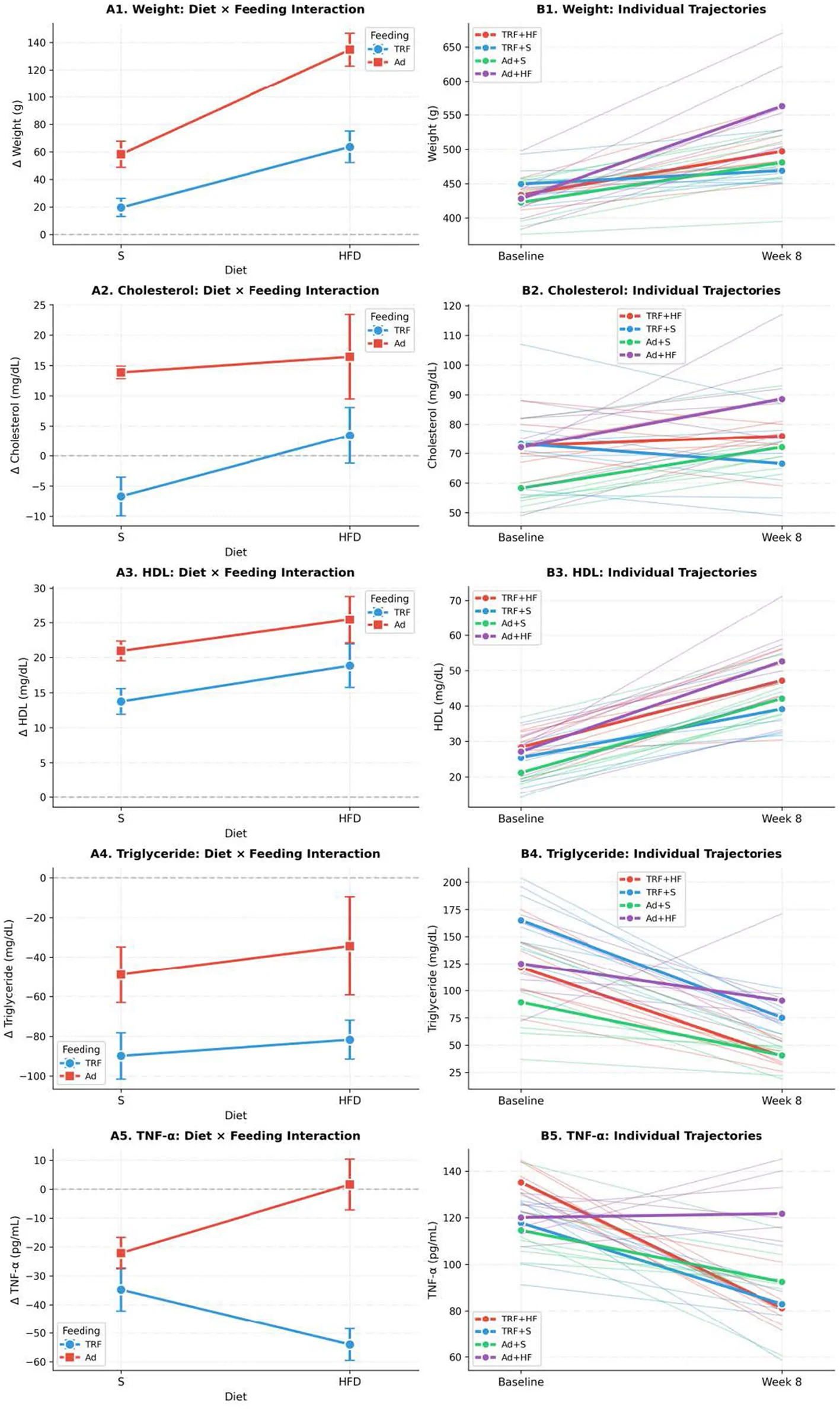
Interaction plots and individual trajectories for variables with significant group × time interactions. Left column **(A1–A5)**: Interaction plots showing mean change scores (Δ) as a function of diet type (x-axis: S, standard diet; HFD, high-fat diet) with separate lines for feeding pattern (TRF, time-restricted feeding; Ad, ad libitum). Error bars represent standard error of the mean. Non-parallel lines indicate diet × feeding interaction effects. Right column **(B1–B5)**: Spaghetti plots displaying individual rat trajectories (thin lines) with overlaid group means (thick lines), illustrating within-group variability and directional changes from baseline to week 8. Selected outcomes shown are body weight **(A1, B1)**, total cholesterol **(A2, B2)**, HDL-C **(A3, B3)**, triglycerides **(A4, B4)**, and TNF-α **(A5, B5)**. Formal inferential results from the baseline-adjusted factorial ANCOVA are reported in Table 4. TRF + H, time-restricted feeding with high-fat diet; TRF + S, time-restricted feeding with standard diet; Ad + S, ad libitum with standard diet; Ad + HF, ad libitum with high-fat diet.

Complementary analyses further showed that triglyceride (grand mean change: −50.8%), insulin (−16.4%), and LPS (−38.5%) decreased across the experimental groups. Group-specific deviations from these overall trends were also observed. For triglycerides, TRF + HF and TRF + S showed deviations of −18.0 and −26.2 mg/dL, respectively, whereas Ad + S and Ad + HF showed deviations of + 14.8 and + 29.4 mg/dL. For TNF-α, Ad + HF showed a positive deviation from the overall trend (+ 28.9 pg/ml). Endpoint variance was consistently higher than baseline variance across biomarkers, with variance ratios ranging from 1.95 to 2.56, indicating greater inter-individual variability after the intervention.

The interaction plots (Figure 4, left column) display mean change scores as a function of diet type, with separate lines for each feeding pattern. For body weight (A1), both high-fat diet and *ad libitum* feeding independently increased weight gain, with the Ad + HF group showing the greatest increase (+ 134.8 g, 31.5%) and TRF+S showing the smallest (+ 19.5 g, 4.3%). The lines diverge toward the high-fat diet end, indicating that *ad libitum* feeding amplifies the weight gain effect of high-fat diet. For cholesterol (A2), *ad libitum* feeding produced increases regardless of diet type, while TRF groups showed minimal change or slight decreases. HDL (A3) increased uniformly across all groups, with parallel lines indicating no substantial interaction. Triglycerides (A4) decreased in all groups, but the magnitude of reduction was substantially greater in TRF groups compared to *ad libitum* groups; TRF + HF showed a 67.2% reduction compared to only 27.4% in Ad + HF. TNF-α (A5) demonstrated the clearest interaction pattern: TRF produced marked reductions regardless of diet (−35 to −54 pg/ml), whereas Ad + HF showed essentially no change from baseline (+ 1.6 pg/ml), resulting in diverging lines that cross between diet conditions. The spaghetti plots (Figure 4, right column) display individual rat trajectories alongside group means, confirming that the observed group differences reflect consistent patterns across individual animals rather than being driven by outliers (Figure 4).

To assess the robustness of findings and address potential regression to the mean effects, supplementary analyses were conducted. Baseline-change correlations revealed significant negative associations for triglyceride (*r* = −0.77, *p* < 0.001), insulin (*r* = −0.69, *p* < 0.001), TNF-α (*r* = −0.57, *p* = 0.002), and LPS (*r* = −0.59, *p* = 0.001), indicating regression to the mean patterns that were controlled through ANCOVA baseline adjustment. Effect size analysis (Cohen's d) demonstrated large within-group effects for TNF-α in TRF + HF (d = −6.15) and TRF + S (d = −2.30), whereas Ad + HF showed negligible change (d = + 0.11). Individual-level examination of Ad + HF revealed that 4 of 7 rats showed TNF-α increases while 3 showed decreases, explaining the near-zero group mean change. Sensitivity analysis excluding one potential outlier in Ad + HF (Rat #3, showing + 137% triglyceride increase) did not alter the significance of interaction effects for triglyceride (*p* < 0.001) or TNF-α (*p* = 0.023), confirming the robustness of primary findings.

## 4 Discussion

The present study demonstrates that time-restricted feeding applied during the inactive phase differentially influenced metabolic outcomes depending on diet composition. The findings indicate that time-restricted feeding limits weight gain and improves inflammatory and lipid profiles, whereas unrestricted high-fat feeding exerts the most detrimental metabolic effects.

Among all measured variables, body weight exhibited the strongest group × time interaction, with *ad libitum* high-fat feeding producing the greatest weight gain, whereas the TRF + S group showed the least increase, indicating a robust protective effect of time-restricted feeding across dietary conditions. Similarly, TNF-α displayed a pronounced interaction effect, characterized by substantial reductions in both TRF groups and a lack of improvement in the Ad + HF group, highlighting the anti-inflammatory potential of time-restricted feeding independent of diet type. Lipid metabolism also showed clear interaction patterns, as total cholesterol increased in both *ad libitum*-fed groups but decreased in the TRF + S group, while triglyceride levels declined most markedly in the TRF + HF group. In contrast, IL-6, LPS, insulin, glucose, and LDL-C did not exhibit significant group × time interactions, suggesting that the metabolic effects of inactive-phase time-restricted feeding are more pronounced for body weight regulation and selected lipid and inflammatory markers than for global glucose–insulin homeostasis. Collectively, these findings indicate that time-restricted feeding limits weight gain and improves inflammatory and lipid profiles, whereas unrestricted high-fat feeding exerts the most detrimental metabolic effects. Importantly, this study was designed to assess the physiological effects of feeding-time manipulation rather than to directly measure circadian rhythmicity.

### 4.1 Body weight

In our study, the body weight findings indicate that restricting the daily eating window during the inactive (light) phase can meaningfully influence weight regulation under both standard and high-fat dietary conditions. Consolidation of food access into a defined daily feeding window, even when scheduled during the light phase, was associated with reduced weight gain compared with *ad libitum* feeding. However, because actual food and energy intake were not quantitatively measured, it is not possible to determine whether this difference resulted from the fasting–feeding schedule itself, lower total energy intake, or both. These findings are consistent with a possible contribution of the prolonged daily fasting interval; however, the present design cannot distinguish the effects of fasting duration from those of feeding during a specific circadian phase. Direct circadian parameters, including activity rhythms, core body temperature, and clock gene expression, were not measured; therefore, circadian mechanisms cannot be directly evaluated in the present study.

Previous studies have shown that time-restricted feeding improves energy balance by narrowing the eating window and enhancing metabolic flexibility (29, 38), with proposed involvement of fasting-related pathways such as AMPK and SIRT1 activation, improved mitochondrial function, and increased lipid oxidation (39). Consistent with previous experimental and mechanistic evidence (38, 40), the reduced weight gain observed in the TRF + HF group relative to the Ad + HF group suggests that prolonged fasting may partially mitigate the obesogenic effects of an HF diet, although the present study cannot distinguish the effects of fasting duration from those of feeding phase.

Rodent studies have consistently demonstrated that TRF attenuates weight gain and metabolic dysfunction when feeding is aligned with the active (dark) phase. For instance, Chaix et al. (41) and Mitchell et al. (40) reported protection against HF diet induced obesity under circadian-aligned TRF. While these studies emphasize the benefits of optimal timing, the present findings extend this evidence by showing that TRF applied during the inactive (light) phase also reduces weight gain relative to *ad libitum* feeding, suggesting that prolonged daily fasting may contribute to the observed effects even when feeding occurs during the inactive phase, although the relative contributions of fasting duration and circadian timing cannot be separated in the present study. Transient weight fluctuations observed in TRF + S at week 6–7 and accelerated gain in Ad + HF after week 6 (see Figure 3) are consistent with established adaptation patterns in rodent dietary studies, where metabolic adjustments typically stabilize after 4–6 weeks (40, 41).

Comparable outcomes have been reported in Ramadan fasting studies, documenting reductions in body weight, fat mass, and waist circumference (34, 42, 43). These effects have been associated with longer daily fasting intervals (12–16 h), changes in total energy intake, and favorable shifts in metabolic hormone profiles such as leptin and adiponectin (44, 45). However, differences in sleep–wake patterns, feeding variability, and behavioral context limit direct translational comparison with the present animal model.

Overall, the consistency between TRF outcomes in rodents and observations from Ramadan fasting in humans suggests that restricting food intake to a defined daily time window can promote metabolic benefits even when eating occurs outside the optimal circadian phase. Although feeding during the active phase may provide additional advantages (46), the present findings cannot determine whether the observed effects were primarily driven by fasting duration, feeding timing, differences in energy intake, or their combination. Future studies incorporating active-phase TRF comparator groups together with behavioral, hormonal, and molecular circadian measurements are required to clarify the relative contributions of these factors.

### 4.2 Inflammatory biomarkers

Restricting food intake to a defined daily window during the inactive (light) phase was associated with a marked reduction in TNF-α, whereas IL-6 and LPS did not show significant Diet × Feeding interactions. Therefore, the present findings support a selective effect on TNF-α rather than a generalized improvement across inflammatory markers. These findings indicate that limiting the duration of daily food exposure, rather than continuous feeding, plays a key role in modulating systemic inflammation.

These observations are consistent with experimental and clinical evidence showing that prolonged daily fasting intervals can reduce systemic inflammation even when food intake is not optimally aligned with the circadian activity phase (27, 29, 38). Fasting has been proposed to activate AMPK and SIRT1 and suppress NF-κB–mediated inflammatory signaling, while enhancing lipid oxidation and autophagy, thereby reducing adipose tissue inflammation (39, 46, 47). In the present study, the marked reduction in TNF-α in both TRF groups suggests that fasting-related metabolic effects may outweigh potential disadvantages of daytime feeding in nocturnal animals, without implying direct circadian regulation.

Previous animal studies applying TRF during the active (dark) phase have consistently reported anti-inflammatory benefits under circadian-aligned conditions. Hatori et al. (46) and Chaix et al. (41) demonstrated reductions in inflammatory signaling and metabolic dysfunction in high-fat–fed mice without reducing caloric intake. While these studies emphasize the benefits of circadian-concordant TRF, the present findings suggest that inactive-phase TRF may influence selected inflammatory pathways, particularly TNF-α, although a broader anti-inflammatory effect was not demonstrated.

These observations are broadly consistent with Ramadan fasting studies reporting decreases in TNF-α, IL-6, and CRP (44, 48, 49). However, differences in sleep–wake patterns, dietary composition, and lifestyle factors limit direct translational comparison. Collectively, human data suggest that fasting-related metabolic and hormonal changes—such as reduced insulin exposure, altered lipid handling, and ketone-mediated suppression of inflammatory signaling—can occur even when food intake is temporally shifted (50–52).

IL-6 responses were modest, with a slight increase in the *ad libitum* standard diet group and stable or mildly reduced levels in both TRF groups. Similar attenuation of inflammatory cytokines has been reported in intermittent fasting studies conducted under different feeding-time conditions, although the magnitude and mechanisms of these effects may vary according to circadian timing (53, 54). LPS levels decreased within both TRF groups; however, because the Diet × Feeding interaction for LPS was not significant, these within-group reductions should not be interpreted as evidence of a TRF-specific effect on metabolic endotoxemia. Chronic high-fat feeding disrupts gut barrier integrity and elevates circulating LPS via TLR4/NF-κB activation (35, 36), and the observed reductions may reflect fasting-related improvements in gut barrier function rather than confirmed circadian–gut axis regulation.

Consistent with this interpretation, Ye et al. reported that TRF mitigated high-fat diet–induced inflammation partly through improvements in gut microbiota composition and hepatic metabolism (55). While circadian restoration was reported in that study, such mechanisms cannot be inferred here due to the absence of microbiota and circadian assessments. Ramadan studies reporting gut microbiota changes (56) further support the role of fasting duration in shaping inflammatory outcomes through context-dependent pathways.

Overall, these findings indicate that inactive-phase TRF was associated with a favorable TNF-α response, while effects on IL-6 and LPS were not clearly attributable to the feeding intervention. Future studies incorporating direct measurements of circadian rhythms, gut microbiota, and tissue-level inflammation are needed to clarify underlying mechanisms.

### 4.3 Insulin and glucose regulation

Insulin levels decreased modestly across all groups, with the largest reduction in Ad + S, while glucose levels were elevated in the Ad + HF group but remained stable in both TRF groups. These results suggest that TRF during the inactive phase can support glucose–insulin regulation through fasting-related mechanisms rather than circadian alignment alone.

Previous studies indicate that TRF improves insulin sensitivity and glucose metabolism independently of caloric restriction. Human studies by Gabel et al. (57) and Sutton et al. (27) demonstrated improved glucose tolerance and fasting insulin with narrowed eating windows despite similar energy intake. In rodents, Chaix et al. (29) showed that TRF alleviated HF diet–induced metabolic disruption through improved hepatic insulin signaling and mitochondrial function. As circadian rhythms were not assessed in the present study, similar clock-dependent mechanisms cannot be confirmed, and the observed effects are more conservatively attributed to fasting-induced metabolic adaptations (38, 58).

Animal studies applying TRF during the active phase have shown stronger improvements in insulin sensitivity and glucose control under circadian-concordant conditions (41, 46). In contrast, the present findings indicate that TRF imposed during the inactive phase can still stabilize glucose levels relative to *ad libitum* HF feeding, despite potential circadian disruption.

Ramadan fasting studies have likewise reported improvements in insulin sensitivity and reductions in fasting glucose (59–61), along with reduced oxidative stress and improved hepatic glucose handling (61, 62). Improvements in HbA1c and insulin function have also been observed in healthy individuals and those with metabolic syndrome (63, 64). However, these studies differ substantially in behavioral and dietary context and are therefore cited for contextual comparison rather than direct equivalence.

Taken together, the present results suggest that extended daily fasting periods can support glucose–insulin homeostasis even when feeding occurs during the inactive phase. These effects are most plausibly explained by fasting-driven metabolic adaptations rather than confirmed circadian realignment. Nonetheless, the modest magnitude of the effects indicates that greater benefits may require longer fasting durations, alternative feeding windows, or alignment with the active phase, as shown in circadian-concordant TRF protocols (65).

### 4.4 Lipid metabolism

Our findings show that lipid metabolism was influenced by both diet type and feeding schedule. Time-restricted feeding during the inactive phase (09:00–17:00) improved lipid profiles under both standard and high-fat dietary conditions. The TRF + HF group exhibited the greatest reduction in triglycerides, while total cholesterol increased in both *ad libitum* groups but declined significantly in the TRF + S group. These results indicate that structured fasting periods can beneficially modulate lipid metabolism without implying restoration of circadian rhythmicity.

THESE observations are consistent with previous studies demonstrating that TRF improves dyslipidemia through enhanced hepatic lipid turnover, increased fatty acid oxidation, and suppression of *de novo* lipogenesis (29, 46). Fasting-induced activation of AMPK and PPARα pathways is known to promote fat oxidation and reduce hepatic triglyceride synthesis (29, 38). As circadian parameters were not assessed, these mechanisms are discussed as established fasting-related pathways rather than circadian effects in this model.

Rodent studies applying TRF during the active (dark) phase have consistently reported improvements in lipid metabolism under circadian-aligned conditions. Hatori et al. (46) and Chaix et al. (41) showed reductions in hepatic steatosis, plasma triglycerides, and total cholesterol in HF–fed mice without reducing caloric intake. While these findings highlight benefits under optimal circadian alignment, the present study extends this evidence by showing that inactive-phase TRF can also confer significant lipid-metabolic benefits.

Ramadan fasting studies further support these findings, reporting favorable changes in lipid profiles (59–61). These effects have been attributed to prolonged daily fasting intervals, enhanced insulin sensitivity, and increased fat mobilization, leading to improved hepatic lipid handling (62, 64).

The pronounced reduction in triglycerides in the TRF + HF group suggests increased utilization of stored lipids, potentially through enhanced lipolysis and reduced hepatic VLDL secretion. In contrast, continuous *ad libitum* feeding—particularly under high-fat conditions—likely promotes sustained hepatic lipid synthesis, whereas fasting intervals facilitate lipid clearance and normalization of circulating triglycerides (65).

Several biomarkers, including triglyceride, insulin, and LPS, exhibited parallel decreases over time across all experimental groups, representing an unexpected background trend. Although this uniform reduction cannot be fully explained within the scope of the present study, maturation-related adaptations of the gut microbiome during the experimental period may have contributed by reducing intestinal LPS translocation, with downstream effects on inflammatory markers and insulin sensitivity across groups. While the mechanisms underlying this global downward trend remain uncertain, treatment-specific deviations from this background pattern were evident. Time-restricted feeding groups demonstrated substantially greater reductions in triglycerides and inflammatory markers compared with *ad libitum* groups. In particular, triglyceride levels decreased by 54–67% in the TRF groups, whereas the Ad + HF group showed a smaller reduction (27%) that did not reach within-group statistical significance. Notably, for TNF-α, the Ad + HF group exhibited a relative increase compared with other groups, consistent with the pro-inflammatory effects of high-fat feeding observed in a subset of animals. These divergent responses, reflected in significant group × time interactions, indicate that experimental conditions differentially modulated metabolic trajectories and support the conclusion that time-restricted feeding confers additional metabolic benefits beyond the shared background trend.

Interpretation of HDL-C changes requires consideration of species-specific lipid metabolism. Rodents lack cholesteryl ester transfer protein activity, resulting in an HDL-dominant lipoprotein profile that limits direct extrapolation to human cardiovascular risk (66). Accordingly, composite indices including non-HDL cholesterol and the TC/HDL ratio were evaluated. Although these indices improved across all groups, no significant effects of diet or feeding pattern were detected, suggesting generalized metabolic adaptation rather than feeding-schedule–specific effects.

Overall, these findings indicate that inactive-phase TRF can meaningfully improve lipid metabolism, most plausibly through fasting-driven hepatic and peripheral adaptations such as enhanced lipid oxidation and improved insulin sensitivity. As circadian markers were not measured, causal inferences regarding circadian mechanisms cannot be made. Future studies incorporating direct circadian and tissue-level assessments are required to clarify the interaction between fasting duration, meal timing, and lipid metabolic regulation.

### 4.5 Limitations

While this study offers promising evidence on the metabolic consequences of inactive-phase time-restricted feeding, several limitations should be considered when interpreting the findings. Most importantly, the design did not include a time-restricted feeding condition aligned with the active (dark) phase. As a result, we cannot directly contrast circadian-aligned vs. circadian-misaligned TRF, nor can we clearly separate the relative contributions of fasting duration, feeding timing, and their interaction. Including both inactive- and active-phase TRF arms in future work will be necessary to disentangle these components.

Food consumption was monitored qualitatively, but it was not systematically quantified in grams. Consequently, total energy intake could not be estimated or included as a covariate in the statistical models. This is an important limitation because the TRF groups had access to food for only 8 h per day, whereas the *ad libitum* groups had unrestricted access. Therefore, we cannot determine whether the observed differences in body weight and metabolic outcomes were attributable to the timing and duration of food access, differences in total caloric intake, or a combination of these factors. Future studies should include systematic measurement of food and energy intake and, ideally, pair-fed comparison groups to distinguish these effects.

Some outcomes also differed at baseline—most notably TNF-α and triglycerides—necessitating baseline-adjusted ANCOVA. In addition, individual responses showed substantial variability, which may have attenuated or amplified certain group-level estimates and should be taken into account when generalizing the magnitude of effects.

Only male rats were studied, selected because they tend to gain diet-induced weight more rapidly within the experimental timeframe. This choice improves feasibility but limits generalizability to females; inclusion of both sexes will be important to evaluate potential sex-specific responses.

The 8-week intervention was sufficient to detect clear between-group differences and moderate-to-large effects, yet it may have limited sensitivity to smaller effects or longer-term metabolic adaptations. Moreover, the study was designed to evaluate fasting-related metabolic changes rather than circadian clock resetting; therefore, we do not infer that circadian rhythms were restored or materially modified.

To standardize metabolic conditions, all animals underwent a 6-h fast before blood sampling. Although shorter than the overnight fasting periods used in some lipid protocols, it was applied uniformly at baseline and endpoint, supporting valid within-group and between-group comparisons under consistent conditions.

Finally, tissue-level endpoints (e.g., hepatic morphology/steatosis, inflammatory infiltration, intestinal permeability, and gut microbiota composition) were not assessed. While circulating biomarkers provided a useful systemic readout, these measures would enable more direct evaluation of organ-specific and gut-mediated pathways. Likewise, direct circadian markers—such as locomotor activity rhythms, core body temperature, or clock gene expression—were not measured, so circadian interpretations remain inferential and grounded in established literature rather than direct experimental confirmation.

## 5 Conclusions

In summary, the present study shows that time-restricted feeding applied during the inactive (light) phase produces clear metabolic effects in a rat model. Inactive-phase TRF was associated with improved body weight regulation, favorable lipid-related changes, and a reduction in TNF-α, whereas no significant intervention-specific effects were observed for IL-6 or LPS. These results indicate that, under the present experimental conditions, a consistent daily fasting–feeding schedule was associated with favorable changes in body weight regulation and selected metabolic outcomes. However, because total food intake was not quantified, the relative contributions of fasting duration, feeding timing, and possible differences in energy intake cannot be determined from the present study.

Nevertheless, as direct circadian measurements and tissue-level mechanistic analyses were not included, the findings should be interpreted as evidence of fasting-driven metabolic adaptation rather than altered or preserved circadian alignment. While circadian timing may modulate the magnitude of these effects, the present study cannot determine the relative contributions of fasting duration, feeding phase, and energy intake. Accordingly, the findings should be interpreted as metabolic responses associated with inactive-phase TRF rather than evidence of circadian realignment or of a dominant fasting-duration effect.

Together, these findings contribute to the growing experimental literature on time-restricted feeding by demonstrating that inactive-phase TRF elicits measurable metabolic benefits in rodents. Future studies incorporating direct circadian markers, activity rhythms, and organ-specific endpoints will be essential to delineate the interaction between feeding time and fasting duration and to define the mechanistic basis of these effects.

## Data Availability

The raw data supporting the conclusions of this article will be made available by the authors, without undue reservation.
