# Correction: Time-restricted feeding during the inactive phase vs. *ad libitum* feeding: diet-dependent effects on weight and metabolic markers in male Wistar rats

**DOI:** 10.3389/fnut.2026.2021620

**Published:** 2026-10-22

**Authors:** Ladan Hajhamidiasl, Murat Baş, Ozgur Yilmaz, Samed Ozer, Salim Yilmaz, Sedat Arslan

**Affiliations:** 1 Department of Nutrition and Dietetics, Faculty of Health Sciences, Acibadem Mehmet Ali Aydinlar University, Istanbul, Türkiye; 2 Department of Nutrition and Dietetics, Graduate School of Health Sciences, Acibadem Mehmet Ali Aydinlar University, Istanbul, Türkiye; 3 Animal Application and Research Center, Acibadem Mehmet Ali Aydinlar University, Istanbul, Türkiye; 4 Department of Healthcare Management, Faculty of Health Sciences, Acibadem Mehmet Ali Aydinlar University, Istanbul, Türkiye; 5 Department of Healthcare Management, Graduate School of Health Sciences, Acibadem Mehmet Ali Aydinlar University, Istanbul, Türkiye; 6 Department of Nutrition and Dietetics, Bursa Uludag University, Bursa, Türkiye

**Keywords:** *ad libitum*, circadian rhythm, high-fat diet, intermittent fasting, time-restricted feeding

Affiliation “Department of Nutrition and Dietetics, Graduate School of Health Sciences, Acibadem Mehmet Ali Aydinlar University, Istanbul, Türkiye” was omitted from the paper. This affiliation has now been added for authors Ladan Hajhamidiasl and Murat Baş.

Affiliation “Department of Healthcare Management, Graduate School of Health Sciences, Acibadem Mehmet Ali Aydinlar University, Istanbul, Türkiye” was omitted from the paper. This affiliation has now been added for author Salim Yilmaz.

Affiliation “Department of Health Management, Faculty of Health Sciences, Acibadem Mehmet Ali Aydinlar University, Istanbul, Türkiye” was erroneously given for author Salim Yilmaz. The correct affiliation is “Department of Healthcare Management, Faculty of Health Sciences, Acibadem Mehmet Ali Aydinlar University, Istanbul, Türkiye.”

The original version of this article has been updated.

